# Application of anatomy unit resection surgery for lateral basicranial surgical approach in oral squamous carcinoma

**DOI:** 10.1186/s12903-023-02708-6

**Published:** 2023-01-07

**Authors:** Kun Wu, Ke-yue Liu, Zhao-jian Gong, Sheng Zhang, Zhen-hu Ren, Han-jiang Wu

**Affiliations:** 1 Department of Oral and Maxillofacial Surgery, Second Xiangya Hospital of Central South University, Renmin Road, No. 139, Changsha, 410011 Hunan China; 2 Department of Oral and Maxillofacial-Head and Neck Oncology, Shanghai Ninth People’s Hospital, Shanghai Jiao Tong University, School of Medicine, No.639, Zhizaoju Road, 200011 Shanghai, China

**Keywords:** Lateral basicranial surgical approach, Anatomy unit resection surgery, Posterior oral squamous cell carcinoma, Overall survival rate

## Abstract

**Background:**

The basicranial region lacks definite boundaries and includes various anatomical units. We developed a novel concept of the posterior oral anatomical complex (POAC) to identify these anatomical units in the basicranial region. OSCC with POAC involvement is termed posterior oral squamous cell carcinoma (POSCC) with poor prognosis. The principal aim of this study was to evaluate the effect of anatomy unit resection surgery (AUSR) on patients with POSCC.

**Methods:**

A total of 120 POSCC patients who underwent radical surgical treatment were recruited for this study. These POSCC patients were treated with conventional surgery or AUSR. According to the extent of primary tumor resection in the AUSR group, the lateral basicranial surgical approach can be subdivided into four types: face-lateral approach I, face-lateral approach II, face-median approach or face-median and face-lateral combined approach. Facial nerve function was evaluated according to the House-Brackmann Facial Nerve Grading System.

**Results:**

The overall survival rate was 62.5% and 37.5% in the AURS group and conventional group (hazard ratio: 0.59; p < 0.0001), respectively. The disease-free survival rate was 62.5% and 34.3% in the AURS group and conventional group (hazard ratio: 0.43; p = 0.0008), respectively. The local disease control rate in the AURS group (71.4%) was significantly better than that in the conventional group (34.4%) in present study (p < 0.0001). Compared to the conventional group, all the patients undergoing AURS were classified as T4 stage and presented with more lymph node metastasis (71.4%). A total of 20 patients (face-lateral approach I and face-lateral combined approach) were temporarily disconnected from the temporofacial branch of the facial nerve. Fifteen patients exhibited slight paresis, and five patients presented with moderate or severe paresis. The survival rate of zygomatic arch disconnection was 94.6% (54 of 56 patients).

**Conclusion:**

This lateral basicranial surgical approach based on AUSR improves the survival rate and enhances the local control rate while also preserving a good prognosis without damaging the nerve and zygomatic bone. This surgical approach based on AUSR provides a novel and effective surgical treatment to address POSCC with better prognosis, especially for patients without metastatic lymph nodes.

## Introduction

Lip and oral cavity cancers are relatively common cancers worldwide, with approximately 350,000 new cases and 170,000 deaths being reported [1]. Histologically, squamous cell carcinoma accounts for more than 90% of malignant lesions in the oral cavity, with a 5-year overall survival rate of approximately 50% [2]. Comprehensive treatment (including surgery, chemotherapy, radiotherapy and other treatments) is universally accepted as the most appropriate management for oral squamous cell carcinoma (OSCC) patients, especially for advanced OSCC patients. Despite continuous therapeutic advancements in the past several years, the overall prognosis has remained poor [3, 4]. Undoubtedly, surgical treatment remains the mainstay of comprehensive treatment [5], and it exerts a considerable effect on the prognosis of OSCC patients [6, 7].

OSCC can be subdivided according to primary tumor sites, such as the tongue, buccal mucosa, maxillary/mandibular gingiva, floor of the mouth and palate. Due to the specific anatomical locations of OSCC, the overall survival rate of different OSCC subtypes can significantly vary [8, 9]. Previous studies have obtained similar conclusions that buccal squamous cell carcinoma (BSCC) has a worse prognosis than other OSCCs due to the high incidence of locoregional recurrence [8, 10]. In addition, differences in TNM stage may account for why BSCC appears to have a worse prognosis than other OSCCs [8]. BSCC has a high incidence of local recurrence, even with pathologically negative margins [9].

**Fig. 1:**
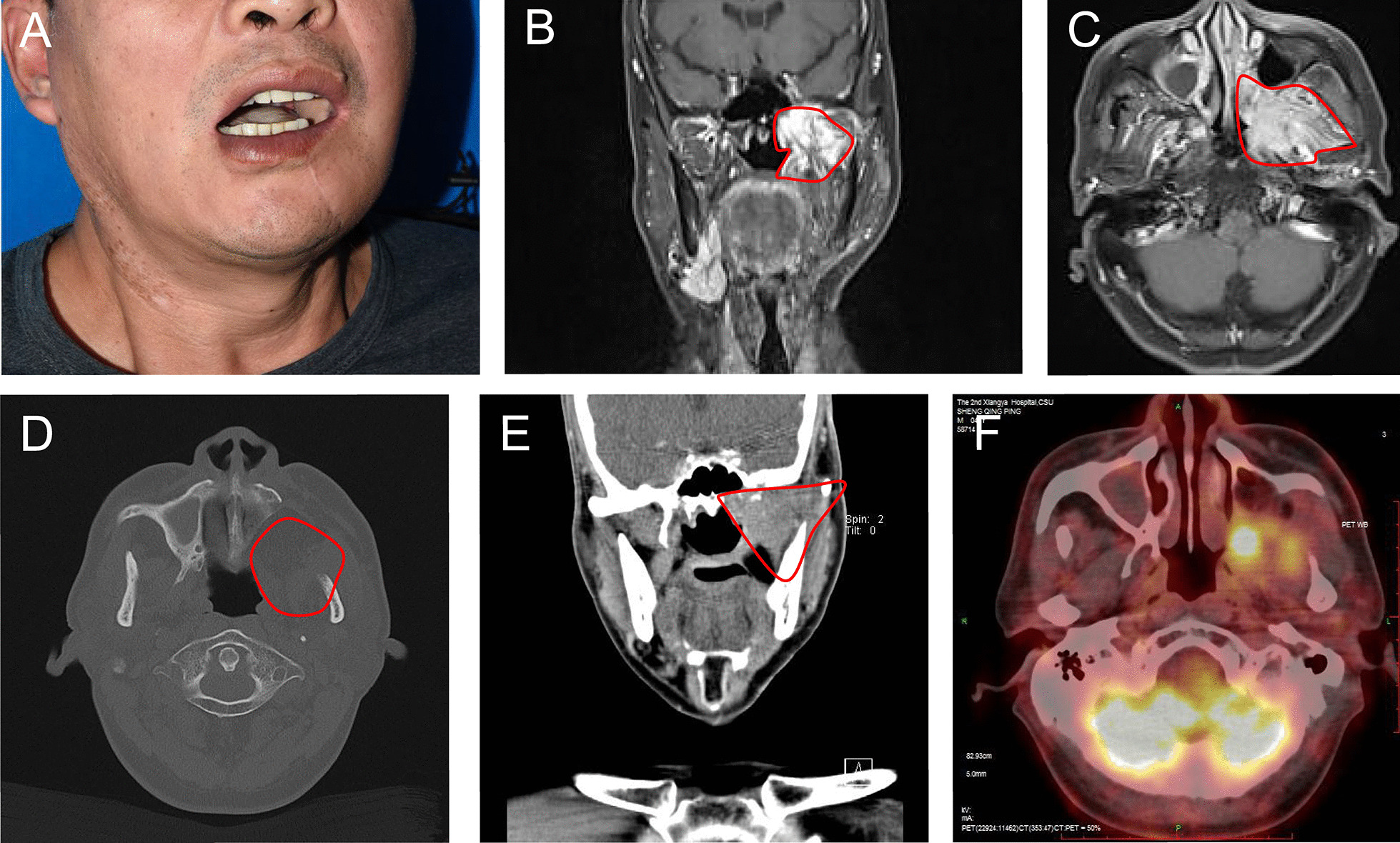
The recurrent POSCC located at basicranial region **A** The photograph of recurrent POSCC patient, **B**, **C** MR indicated that the recurrent tumor involved POAC, **D**, **E** CT indicated that bone destruction was demonstrated in mandibular ramus, infratemporal surface of maxillary body and pterygoid process, **F** PET-CT suggested the recurrent tumor located at basicranial region

**Fig. 2:**
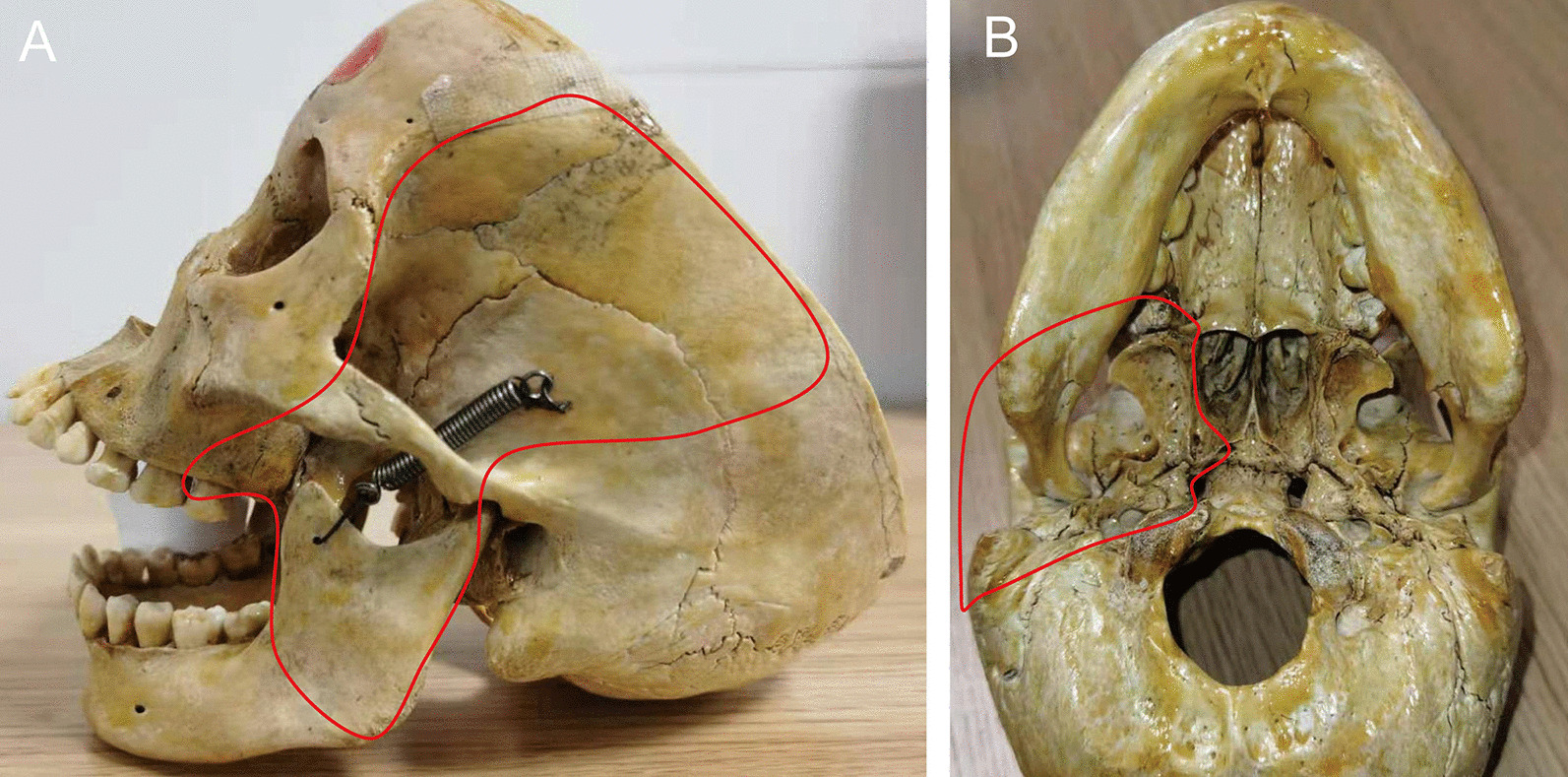
The boundary of POAC **A** The POAC’s boundary viewed from side face, **B** The POAC’s boundary viewed from skull base

For posterior BSCC, we clinically observed that almost all recurrent tumors were located in the basicranial region and involved surrounding anatomical units (Fig. 1). The characteristic feature of the basicranial region is the absence of definite boundaries and the inclusion of various anatomical units. We developed a novel concept of the posterior oral anatomical complex (POAC) to identify these anatomical units, which consists of the posterior mandibular body, mandibular ramus, infratemporal surface of the maxillary body, pterygoid process, temporalis, medial pterygoid and lateral pterygoid (Fig. 2). Due to the indefinite anatomical boundaries and surrounding space of POAC, tumor cells can spread along muscle fibers and their surrounding spaces to the basicranial region [11]. OSCC with POAC involvement is known as posterior oral squamous cell carcinoma (POSCC). In our previous study, we found that anatomy unit resection surgery (AUSR) involves the removal of the entire anatomical unit (or subunit) with tumor involvement and significantly improves the overall survival rate in BSCC patients [11, 12]. Therefore, the principal aim of this study was to evaluate the effect of AUSR on patients with POSCC.

## Materials and methods

### Study design

This study was a retrospective cohort study. A total of 120 POSCC patients who underwent radical surgical treatment were recruited from March 2014 to December 2019 in the Department of Oral and Maxillofacial Surgery at the Second Xiangya Hospital, Central South University. These POSCC patients were treated with conventional surgery or AUSR. POSCC patients with a history of surgical treatment, chemotherapy or radiotherapy were excluded from the analyses. The study was approved by the institutional review board of the Second Xiangya Hospital (Approval Number: 2011210), and informed consent was obtained from all of the participants. The study was performed in accordance with the Declaration of Helsinki.

All of the patients were pathologically and preoperatively diagnosed with squamous cell carcinoma. Head and neck magnetic resonance imaging (MRI), head and neck computed tomography (CT) and PET-CT were used to evaluate the location and size of the POSCC primary tumor and cervical lymph nodes. For these patients, the preoperative imaging examination and clinical examination indicated the primary tumor with POAC involvement.

#### Facial nerve function evaluation criteria

Facial nerve function was evaluated according to the House–Brackmann Facial Nerve Grading System. Patients with a score between 5 and 10 points were identified as having normal or slight paresis, and patients with a score between 11 and 30 points were identified as having moderate, severe or total paresis [13].

### Surgical technique

In the conventional surgery group, the universally accepted standard treatment was performed with a wide resection of the primary tumor with larger free macroscopic margins (1.5–2 cm)[14]. In the AUSR group, the entire anatomical unit (or subunit) with tumor involvement was completely removed. According to the extent of tumor resection, AUSR for the lateral basicranial surgical approach can be subdivided into four types.

### Face-lateral approach I

#### Indication

Posterior BSCC (stage T4, penetrating resection) with limited mouth opening and involving the mandibular ramus, maxillary tuberosity, medial and lateral pterygoid plate, medial pterygoid, lateral pterygoid, temporalis and masseter.

#### Incision design

**Fig. 3:**
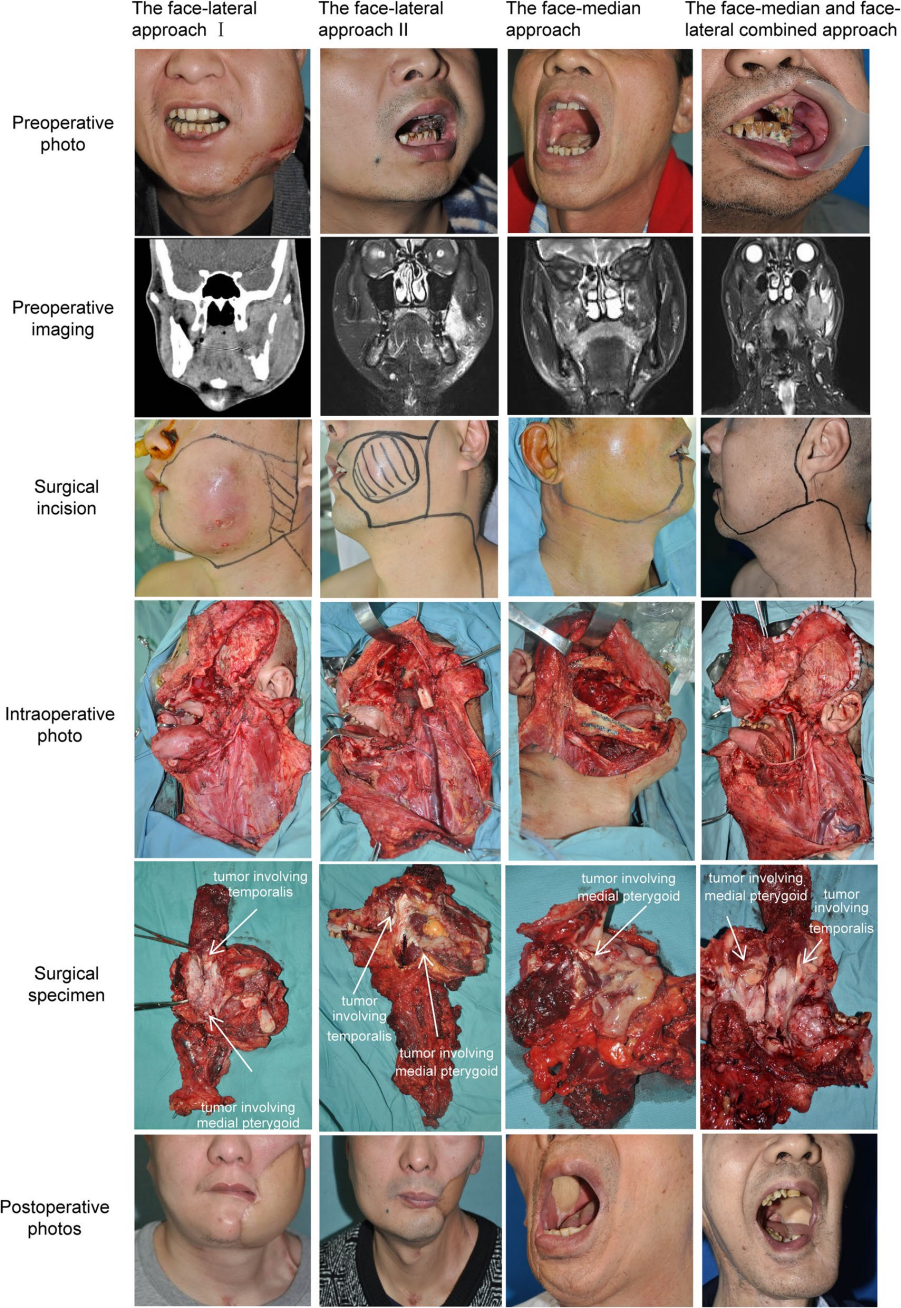
Four types of lateral basicranial surgical approach According to the extent of tumor resection, the AUSR for lateral basicranial surgical approach could be subdivided into the four types: face-lateral approach I, face-lateral approach II, face-median approach or face-median and face-lateral combined approach

The incision was made to perform a penetrating resection of BSCC and to extend upward to the temporoparietal region (Fig. 3). Subsequently, the zygomatic arch, the zygomatic bone and the temporofacial branch of the facial nerve were disconnected. The zygomatic arch, zygomatic bone and temporofacial branch of the facial nerve were restored after removing the primary tumor.

#### Excision extension

Primary tumor (involving the skin); parotid; portions of maxilla and mandible; buccal fat pad; medial and lateral pterygoid plate; posterior buccinator; origins and terminations of masseter, temporalis, medial pterygoid and lateral pterygoid.

### Face-lateral approach II

#### Indication

Posterior BSCC (stage T4, penetrating resection) invading upward and not extending beyond the level of the zygomatic arch.

#### Incision design

The incision was made to perform a penetrating resection of BSCC and to extend upward to the temporal region (Fig. 3). Subsequently, the zygomatic arch and the zygomatic bone were disconnected. Furthermore, the temporofacial branch of the facial nerve was dissected and retained via upward traction.

#### Excision extension

Primary tumor (involving the skin); parotid; portions of maxilla and mandible; buccal fat pad; medial and lateral pterygoid plate; posterior buccinator; origins and terminations of masseter, medial pterygoid and lateral pterygoid; the portion of the temporalis under the eyebrow bow level.

### The face-median approach

#### Indication

Posterior BSCC (stage T4, nonthrough resection); central carcinoma of the jaw centered on the mandibular molar region (involving the mandibular ramus); gingival cancer centered on the mandibular molar region (involving the mandibular ramus); gingival cancer centered on the maxillary molar region (involving the maxillary tuberosity and pterygoid plates) and oropharyngeal cancer (involving the medial pterygoid muscles or maxillary tuberosity).

#### Incision design

The incision started from the middle of the lower lip or the mouth corner (wheter retain nervus mentalis), went along with the mentolabial sulcus to the skin of the neck (2 cm below the jawbone) and lengthened to the earlobe, extending downward to the clavicle and upward to the temple. To completely remove the POAC and primary tumor, an intraoral incision was performed along the maxillary gingival margin to the media line. The face-cheek flap was developed from the superficial level of the masticatory muscles. Afterwards, the incision extended to the level brow arch to temporarily cut off the zygomatic arch and ranged to the deep temporal fat pad (Fig. 3).

#### Excision extension

The primary tumor; a portion (or half) of the maxilla and mandible; medial and lateral pterygoid plate; posterior buccinator; buccal fat pad; buccopharyngeal fascia; origins and terminations of masseter, medial pterygoid and lateral pterygoid; the portion of the temporalis under eyebrow bow level.

### The face-median and face-lateral combined approach

#### Indication

Posterior BSCC (stage T4, nonthrough resection); central carcinoma of the jaw centered on the mandibular molar region (involving the mandibular ramus); gingival cancer centered on the mandibular molar region (involving the mandibular ramus); gingival cancer centered on the maxillary molar region (involving the maxillary tuberosity and pterygoid plates) and oropharyngeal cancer (involving the medial pterygoid muscles or maxillary tuberosity). The imaging results showed that the tumor invaded upward and extended beyond the level of the zygomatic arch.

### Incision design

The incision started from the middle of the lower lip or the mouth corner, went along with the mentolabial sulcus to the skin of the neck (2 cm below the jawbone) and lengthened to the earlobe, extending downward to the clavicle and upward to the temporoparietal region. The intraoral incision was performed along the maxillary gingival margin to the media line. The face-cheek flap was developed from the superficial level of the masticatory muscles. Subsequently, the zygomatic arch and the temporofacial branch of the facial nerve were temporarily disconnected to reach the deep temporal fat pad (Fig. 3). The zygomatic arch and the temporofacial branch of the facial nerve were restored after removing the primary tumor.

### Excision extension

The primary tumor; a portion (or half) of the maxilla and mandible; parotid; medial and lateral pterygoid plate; posterior buccinator; buccal fat pad; buccopharyngeal fascia; origins and terminations of masseter, medial pterygoid, lateral pterygoid and temporalis.

All of the POSCC patients underwent reconstruction with an anterolateral thigh flap and tracheotomy. The disconnected temporofacial branch of the facial nerve was performed with microsurgery. The free zygomatic arch was restored and fixed with titanium plates (Biomet Microfixation, Inc., USA).

### Statistical analysis

Data were analyzed by using SPSS 19 (SPSS, Inc., Chicago, IL, USA). The significance of the differences between the groups was determined by using the t test, Fisher’s exact test or chi-squared test, according to the types of data and distribution. Overall survival, disease-free survival and local disease control rate were analyzed from the date of surgical treatment to the date of event or latest follow-up. Death was identified as a competing event. Moreover, survival experience was analyzed by using Kaplan‒Meier curves. All of the tests were two-sided, and statistical significance was set at p < 0.05.

## Results

**Fig. 4:**
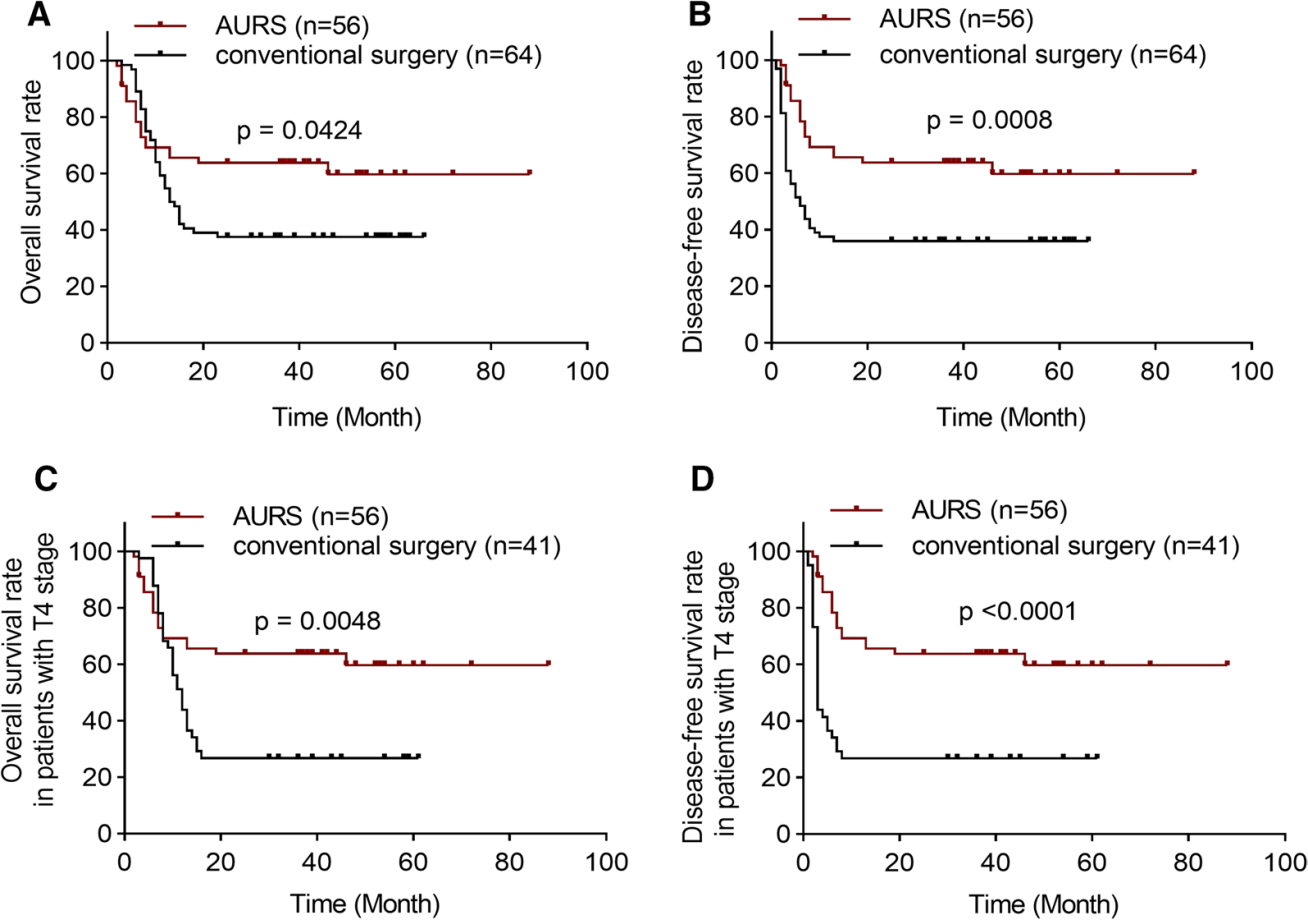
The OS and DFS in AURS and conventional groups **A**, **B** The OS and DFS curve of AURS and conventional groups, **C**, **D** The OS and DFS curve of patients with T4 stage in AURS and conventional groups

Among the patients, 114 patients were male, and 6 patients were female. The mean age of the patients was 49.8 years (range: 28–72 years). The clinical characteristics are presented in Table 1. The primary tumors of all of the patients in the AURS were classified as T4 stage. The T stage (p < 0.0001) and N stage (p = 0.0168) in the AURS group were more advanced than those in the conventional group. The size of the free flap (p < 0.0001) and the duration of operation (p < 0.0001) in the AURS group were significantly increased compared with those in the conventional group. In addition, there were no significant differences in other clinical characteristics between the control and experimental groups. The duration of follow-up ranged from 1 to 88 months, with a 100% follow-up rate. The overall survival rate (OS) was 62.5% and 37.5% in the AURS group and conventional group (hazard ratio: 0.59; p < 0.0001), respectively (Fig. 4A, Table 1). The disease-free survival rate (DFS) was 62.5% and 34.3% in the AURS group and conventional group (hazard ratio: 0.43; p = 0.0008), respectively (Fig. 4B, Table 1). Moreover, the local disease control rate in the AURS group was significantly higher than that in the conventional group (p < 0.0001) (Table 1). Upon further analysis, the OS and DFS of the patients (n = 41) with T4 stage in the conventional group was 26.8% (p = 0.0048) and 26.8% (p < 0.0001), respectively, which was dramatically decreased compared to that in the AURS group (Fig. 4C and D). Interestingly, the DFS of patients with metastatic lymph nodes or without metastatic lymph nodes was 50% (20 of 40 patients) or 93.7% (15 of 16 patients) in the AUSR group, respectively.

**Table 1:** Clinical characteristics of patients treated with AURS or compartment surgery

Group	No. Of patients (%)		p value
	AURS (n = 56)	conventional surgery (n = 64)	
Age(y)	51.2 ± 8.6	48.66 ± 8.893	0.1086
Gender			0.4159
Man	52 (92.9)	62 (96.9)	
Woman	4 (7.1)	2 (3.1)	
Neck dissection			0.0057
Radical	62 (94.7)	49 (76.6)	
Functional	2 (5.3)	15 (23.4)	
T status			
T2	0 (0)	21 (32.8)	< 0.0001
T3	0 (0)	2 (3.1)	
T4	56 (100)	41 (64.1)	
N status			
N ( −)	16 (28.6)	32 (50)	0.0168
N ( +)	40 (71.4)	32 (50)	
Preexisting disease			
Hypertension	5 (8.9)	7 (10.9)	0.4285
Diabetes mellitus	1 (1.8)	4 (6.3)	
Re-exploration			
Yes	0 (0)	2 (3.1)	0.1822
No	56 (100)	62 (96.7)	
Smoking history			
Yes	45 (80.3)	59 (92.2)	0.0572
No	11 (19.7)	5 (7.8)	
Alcohol history			
Yes	40 (71.4)	54 (84.4)	0.0859
No	16 (28.6)	10 (15.6)	
Areca-nut history			
Yes	41 (73.2)	48 (75)	0.8236
No	15 (26.8)	16 (25)	
Flap size (cm^2^)	138.4 ± 65.4	81.6 ± 39.5	
Type of free flap			< 0.0001
ALT		62 (96.9)	0.1822
Fibula free flap	56 (100)	2 (3.1)	
Post-operative PI	0 (0)		
Yes		5 (7.8)	0.544
No	7 (12.5)	59 (92.2)	
Post-operative Length of stay	49 (87.5)	10.2 ± 3.3	
Duration of operation		8.3 ± 2.1	0.0612
Overall survival rate	11.6 ± 4.6	24 (37.5)	< 0.0001
Disease-free survival rate Local disease control rate	11.1 ± 1.6 35 (62.5) 35 (62.5) 40 (71.4)	22 (34.3) 22 (34.4)	0.0424 0.0008 < 0.0001

The data showed as mean ± SD

*ALT* anterolateral femoral free flap; *PI* pulmonary infection

In the AURS group, the face-lateral approach I, face-lateral approach II, face-median approach or face-median and face-lateral combined approach were 18, 15, 21, or 2, respectively. A total of 20 patients (face-lateral approach I and face-lateral combined approach) had the temporofacial branch of the facial nerve disconnected. Fifteen patients presented with slight paresis, and five patients presented with moderate or severe paresis. Furthermore, the survival rate of the disconnected zygomatic arch was 94.6% (54 of 56 patients).

## Summary of the lateral basicranial surgical approach

### The advantages and disadvantages of each lateral basicranial surgical approach

#### Face-lateral approach

The most significant advantage of the face-lateral approach is to obtain enough exposure in the operation field, whereas the disadvantages include atrophy of the temporal fat pad and temporary or permanent facial paralysis.

#### Face-median approach

The advantage of the face-median approach was that it was not damaging to the facial nerve; additionally, it did not elicit atrophy of the temporal fat pad. The exposure of the operation field was worse in the face-median approach than in the face-lateral approach.

#### Face-median and face-lateral combined approach

The operation field of the face-median and face-lateral combined approach was fairly visible. The face-median and face-lateral combined approach requires a temporary disconnection of the temporofacial branch of the facial nerve and may lead to skin necrosis in the middle of the lower lip or at the mouth corner.

### The key points of the lateral basicranial surgical procedure

#### Exposure of the lateral basicranial region

The temporofacial branch of the facial nerve was dissected and disconnected (or retracted). After cutting off the zygomatic arch, the temporalis was resected to reach the temporal bone surface and dissect downward to the lateral basicranial region.

#### The resection of POAC

The mandible was cut off on the mesial side and disconnected from the mandibular posterior margin and deep medial pterygoid to condylar. Afterwards, the adherent soft tissue of the temporal-mandibular joint was disconnected to dissociate the condylar (retain or resect articular disk). Subsequently, the posterior portion of the maxillary was resected, and the medial and lateral pterygoid plates were cut off on the basilar plane (from back to front). Finally, the lesions, including POAC, primary tumor and neck dissection tissue, were completely removed.

#### Control of hemorrhage in the lateral basicranial surgical approach

Ligation of the external carotid artery or maxillary artery was performed before resection of the primary tumor. In resection of the POAC and primary tumor, excision of the medial and lateral pterygoid plate was the final step.

### The reconstruction of POSCC patient defects:

The anterolateral thigh free flap was used to reconstruct complex defects, and fat flaps and muscle tissues of various sizes were harvested to fill the dead spaces.

## Discussion

The oral cavity boundary comprises the lips anteriorly, the mouth floor inferiorly, the cheeks laterally, the oropharynx posteriorly and the palate superiorly [15, 16]. The bone tissue bases of the surrounding muscle tissue are attached to the mandible and maxillary areas. Due to the complexities of oral cavity anatomy, it is not easy to obtain adequate and safe resection margins for OSCC [17]. Previous studies have indicated that OSCC involving POAC can lead to a high locoregional recurrence rate and poor prognosis [18, 19]. To address this challenge, the present study aimed to develop a novel AURS for POSCC to improve treatment options for these patients.

The study by Margaret showed that the local recurrence incidence of T4 stage BSCC was 75% [20]. In this study, the OS and DFS of the patients with T4 stage in the conventional group was 26.8% and 26.8%, respectively, which is similar to the rates of a previous study. For POSCC patients treated with a traditional surgical approach, the OS and DFS were 39.6% and 37.5%, respectively [11]. Although all of the POSCC cases were classified as T4 stage and 71.4% of patients had metastatic lymph nodes in this study, the OS and DFS were 62.5% and 62.5% in the AURS group, respectively, which were dramatically higher than those in the conventional group. These results indicated that AURS is an effective approach to address patients with POSCC.

In POSCC patients, the tumor cells can spread along the muscle fiber and its surrounding space to the basicranial region, which may barely be determined by resection margins [20]. Similarly, the local disease control rate in the AURS group was significantly better than that in the conventional group in the present study. The findings indicated that the application of AUSR could provide a new and effective strategy for POSCC surgical treatment.

The presence of cervical lymph node metastasis is universally accepted as an important prognostic factor in patients with OSCC [21, 22]. In this study, the DFS of patients with metastatic lymph nodes (50%) decreased dramatically compared to those without metastatic lymph nodes (93.7%) in patients treated with AURS. The aggressiveness of tumor cells may explain the difference in DFS between the two groups. Metastatic lymph nodes serve as a key factor in determining the prognosis of POSCC patients [23]. The only patient without metastatic lymph nodes died of contralateral metastatic lymph nodes. None of these patients without metastatic lymph nodes had recurrence of the primary site tumor in the AURS group. Moreover, these findings indicated that the application of AUSR is an ideal method to completely resect the primary tumor of POSCC, especially for patients without metastatic lymph nodes.

In this study, the AUSR for the lateral basicranial surgical approach was classified as face-lateral approach I, face-lateral approach II, face-median approach or face-median and face-lateral combined approach, which is dependent on the extent of tumor resection. For tumors extending beyond the level of the zygomatic arch, an incision was made in the temporoparietal region to completely remove the temporalis anterior. For nonthrough resection POSCC, the face-median approach was performed [24]. Compared with the face-median approach, the face-lateral approach obtained enough exposure in the operation field; however, it is not ideal for the protection of the facial nerve and temporal fat pad. The anterolateral thigh free flap is preferred for filling the absence of muscle or soft tissue, which is particularly significant in facial depression resulting from the loss of tissue inside of the zygomatic arch. The donor site of anterolateral thigh free flaps includes abundant soft tissue and various types of flaps, such as chimeric, adipofascial, musculocutaneous and folded flaps [25]. Free flaps are preferred to reconstruct complex defects and fill dead spaces in head and neck defects [24].

Due to the difficulty and complexity of AUSR in POSCC patients, the flap size of the anterolateral thigh flap and duration of operation in the AUSR group were significantly increased compared with those in the conventional group. Similarly, the postoperative length of stay in the AUSR group was also longer than that in the conventional group. To completely remove the temporalis anterior, it is necessary to extend the incision upward to the temporoparietal region and to temporarily disconnect the temporofacial branch of the facial nerve. Facial paralysis is an inevitable complication for patients who undergo complete resection of the temporalis. The study by Eaton showed that 79% of patients with facial nerve anastomosis had some function, and the mean time to recovery was 7 months [26]. For lateral basicranial surgery, most studies have demonstrated at least 50% grade III postoperative recovery, and the duration of nerve palsy was a prognostic factor for postoperative recovery [27]. Our findings suggested that 75% of patients presented with slight paresis, and 25% of patients presented with moderate or severe paresis. This may be explained by the fact that the repair of the facial nerves was immediately performed after tumor extirpation, with a short interval time, thus leading to a good prognosis. Biological and mechanical factors play an important role in bone graft incorporation [28]. There is a consensus that essential techniques for successful bone grafts include adequate graft fixation without movement, the absence of infection and extensive soft tissue coverage [28, 29]. A previous study showed that the success rate of nonvascularized bone grafts for mandibular reconstruction was 87.6%. Our results indicated that the survival rate of disconnected zygomatic arches was 94.6%. The high success rate of free zygomatic arch grafts could be explained by the following factors: 1. the relatively small size of the graft; 2. the absence of dead spaces filled with anterolateral thigh free flaps; and 3. the presence of sufficient tension-free soft tissue. Furthermore, the clinical results of this study showed that patients in the AUSR group had better nerve function and a good prognosis after surgery.

This clinical study had several limitations. The study was a retrospective study with a limited sample size and biases in sample information. Moreover, it lacks the support of a large number of samples and a multicenter prospective study, which is another shortcoming. Further research and evaluation will be conducted in the future.

## Conclusion

This lateral basicranial surgical approach based on AUSR improves the survival rate and enhances the local control rate while preserving a good prognosis without damaging the nerve and zygomatic bone. This surgical approach based on AUSR provides a novel and effective surgical treatment to address POSCC with better prognosis, especially for patients without metastatic lymph nodes.

## Data Availability

The datasets generated and analysed during the current study are not publicly available due the sensitive nature of questionnaire information for the study community but are available from the corresponding author on reasonable request.
